# The Bogert effect revisited: Salamander regulatory behaviors are differently constrained by time and space

**DOI:** 10.1002/ece3.4590

**Published:** 2018-11-22

**Authors:** Vincent R. Farallo, Rebecca Wier, Donald B. Miles

**Affiliations:** ^1^ Department of Biological Sciences Ohio University Athens Ohio; ^2^ Department of Biological Sciences Virginia Tech Blacksburg Virginia; ^3^ San Juan College, College Boulevard Farmington New Mexico

**Keywords:** amphibian, climate change, plethodontid, salamander, thermoregulation, water loss

## Abstract

The use of behavior to buffer extreme environmental variation is expected to enable species to (a) extend the breadth of environments they inhabit beyond that predicted from climatic data and (b) diminish the negative effects of broad scale and chronic disturbances such as climate change. The term Bogert effect refers to behavioral compensation entailing microhabitat selection to maintain performance across a gradient of environmental conditions resulting in evolutionary inertia of physiological traits. Here, we compare microhabitats used by plethodontid salamanders distributed along an elevational gradient to determine whether there is behavioral compensation that buffers them from deleterious temperatures and moisture levels. Overall, salamanders preferred cooler and more mesic environments and occupied microhabitats that maintained constant moisture conditions at both high‐ and low‐elevation sites. Our results suggest that salamanders use microhabitats to regulate temperature and moisture levels, which is consistent with the Bogert effect. Maintenance of more moist conditions may help buffer these species from rising temperatures but only in suitable high‐elevation environments that are likely to disappear over the next century. We conclude that behavioral regulation of temperature and moisture is a potential mechanism for the Bogert effect in plethodontid salamanders.

## 1 INTRODUCTION

Determining the ability of a species to persist in fluctuating environments is a critical goal for biologists (Sekercioglu, Schneider, Fay, & Loarie, 2008; Thomas, 2010), and a central imperative in light of contemporary global climate change (Huey et al., 2012; Williams, Shoo, Isaac, Hoffmann, & Langham, 2008). A species’ sensitivity to environmental change depends, in part, on whether its ecological and physiological requirements remain available under changing abiotic and biotic conditions. Other factors influencing climatic sensitivity include the duration and magnitude of environmental fluctuations, the reliability of environmental cues for changing conditions, and the fitness costs of coping with novel conditions (Chevin, Lande, & Mace, 2010; Ghalambor, McKay, Carroll, & Reznick, 2007; Marais & Chown, 2008; Phillips et al., 2016).

Nonetheless, species are not always at the mercy of environmental conditions. Rather, through the use of behavior, organisms can modify their use of substrates or microhabitats to buffer themselves from extreme conditions (Bogert, 1949; Cowles & Bogert, 1945). Under this process, termed the Bogert effect (Huey, Hertz, & Sinervo, 2003) regulatory behaviors such as thermoregulation shield organisms from selection, thus precluding physiological evolution. For example, tropical *Anolis* lizards maintain constant daytime body temperatures across elevation through thermoregulation but cannot behaviorally buffer the cold at night. Consequently, lower physiological limits evolved faster than upper limits in this group (Muñoz et al., 2014).

Whereas the evolutionary consequences of the Bogert effect are clear, the behavioral mechanisms by which it occurs remain less explored. If a species uses behavioral compensation to buffer changes in environmental conditions, then we expect that individuals in different parts of that species range should exhibit a shift in microhabitat selection to attain optimal levels of performance. A shift in microhabitat use would entail changes in spatial patterns of habitat exploitation, the timing of daily activity, or both. Thus, testing for behavioral compensation affecting physiological performance requires a study design that can detect spatial and temporal changes in microhabitat use. This process is especially enigmatic in amphibians such as salamanders, which are highly vulnerable to variation in environmental moisture as well as temperature (Brattstrom, 1979). Although accumulating evidence demonstrates that rising environmental temperatures are already affecting most terrestrial environments, the impacts of changes to moisture levels have received less attention. Thus far, empirical studies support the reorganization of precipitation regimes (Brown, Valone, & Curtin, 1997; Felzer & Heard, 1999) and rising evaporation (Trenberth, 2011) as the two major moisture‐related outcomes of climate change with some regions expected to experience wetter conditions, but other localities are becoming drier (Trenberth, 2011). Hence, disentangling the ways in which organisms can simultaneously respond to changes in temperature and moisture remains a key challenge.

Thermoregulation in wet‐skinned organisms differs from reptiles, because of constraints for activity imposed by strict moisture requirements. For instance, the Green Anole (*Anolis carolinensis*) has a cutaneous resistance to evaporative water loss of 196 s/cm. Yet, a similarly sized Mountain Dusky Salamander (*Desmognathus ochrophaeus*) has a skin resistance of 0.9 s/cm (Lillywhite, 2006; Spotila & Berman, 1976). This high hydric sensitivity confers a greater cost to activity for amphibians in warming environments compared to other ectotherms. Although often categorized as thermal conformers (Feder, 1983; Moore & Sievert, 2001), amphibians can nonetheless use behavioral compensation to exploit favorable moisture microhabitats, which may also play a key role in how species are impacted by climate change (Riddell & Sears, 2014, 2015 ).

Lungless salamanders (Family: Plethodontidae) rely on cutaneous respiration, which requires moist skin for gas exchange (Whitford, 1973). Thus, plethodontid salamanders are especially sensitive to rising temperatures and drier conditions. Previous studies have demonstrated the interactive effects of water balance and thermoregulation on locomotor performance in frogs and toads (Miller, 1982; Preest & Pough, 1989; Titon, Navas, Jim, & Gomes, 2010; Tracy, Christian, O'Connor, & Tracy, 1993), and these parameters may also set the limits to activity in plethodontid salamanders as well. Hence, behavioral compensation for a salamander species would entail selecting microhabitats with both favorable temperatures and moisture conditions throughout its range. Plethodontid salamanders exploit a variety of terrestrial microhabitats including cover objects on the forest floor, for example, rocks and woody debris, within the leaf litter, and arboreal habitats (Jaeger, 1978; McEntire, 2016; Niemiller, 2005; Regester & Samoray, 2002; Trauth, McCallum, Ball, & Hoffman, 2001). Many species also exist along elevational gradients allowing for behavioral or physiological adaptation to distinct temperature and moisture extremes.

Although plethodontid salamanders have strict physiological requirements for temperature and moisture, they may be able to modify their use of different microhabitats to buffer against extreme conditions. Recent data suggest that some species may thermoregulate through shifts in microhabitat use (Camp, Wooten, Jensen, & Bartek, 2013; Farallo & Miles, 2016). Changes in subsurface environments, that is, vertical movement within the soil, are also common (Caldwell, 1975; Caldwell & Jones, 1973; Grizzell, 1949; Hoff, 1977; Seebacher & Alford, 2002; Vernberg, 1953), which may further serve to buffer against abiotic extremes.

In this study, we empirically test a behavioral mechanism for the Bogert effect along both moisture and temperature gradients in plethodontid salamanders. Specifically, we determined whether, (a) temperatures of microhabitats used by salamanders differ from null model salamanders, indicating behavioral thermoregulation, (b) microhabitats differ in their moisture and temperature characteristics across elevation, and (c) salamanders preferentially utilize microhabitats that permit them to maintain consistent moisture and temperature levels.

## 2 MATERIALS AND METHODS

### 2.1 Field sites

We selected seven sites between May and July 2015 to assess microhabitat variation in temperatures and moisture conditions available to salamanders. We established sites at four high‐elevation localities (1,115–1,515 m) and three at low‐elevation localities (624–846 m). All sites (~100 m^2^) were situated within the Great Smoky Mountains National Park (GSMNP) in North Carolina and Tennessee. Habitats within the GSMNP support one of the highest salamander species richness in the world, which makes our study area an ideal location for assessing how climate change will impact plethodontid salamanders.

### 2.2 Salamander surveys

We completed exhaustive surveys for salamanders in 5 m^2^ plots within the sites. The survey plots were chosen by walking a random distance (1–20 m) in a random compass direction (1–360°) from the center of the site. At least two people completed each survey by starting on opposite sides of the plot and worked toward the center. We searched for salamanders by flipping over all cover objects and scouring the leaf litter. Once a salamander was detected, we recorded species, its capture location, and microhabitat type. We kept salamanders in separate containers until the end of the survey, upon which the individual were released at the point of capture. We conducted a total of 126 surveys, which occurred throughout the day and night hours. Our earliest morning survey was completed at 7:15 a.m., and our latest nocturnal survey was completed at 12:44 a.m. This sampling protocol provided a comprehensive assessment of microhabitat use by salamanders during their normal active (nocturnal) and inactive (diurnal) periods each day. We used the salamander capture locality data to partition our plots into presence and absence categories for each survey period. Therefore, if salamanders were only captured under woody debris during a survey, we used the temperature and water loss data (described below) from under woody debris as our presence microhabitat data, and data from all other microhabitats to represent absence microhabitat temperature and water loss rates.

### 2.3 Measuring temperature and moisture conditions

At each site, we selected microhabitats (*n* = 17–24) commonly used by plethodontid salamanders for characterizing temperature and moisture conditions. This was repeated twice at each site for a range of 38–45 microhabitats per site. We divided these microhabitats into six categories, which we also used to quantify the microhabitats salamanders were using during our surveys (a) above ground, which includes microhabitats at ground level exposed to the air (e.g., above leaf litter, coarse woody debris), (b) tree/shrub which includes animals captured at elevated microhabitats (where we expected an increase in airflow), (c) under woody cover, (d) under rock, (e) under moss, and (f) under leaf litter. We assessed potential water loss rates across the microhabitat categories using agar models of salamanders. Numerous studies have demonstrated inter‐ and intraspecific variation in water loss rates (Peterman, Locke, & Semlitsch, 2013; Riddell & Sears, 2015; Winters & Gifford, 2013). However, to understand the extent to which salamanders use regulatory behaviors to buffer moisture variation, we require information about potential water loss rates across different microhabitat types. Thus, we used agar models to characterize the moisture conditions of microhabitats in each sampling locality. Because these models are porous, microhabitats where agar models lose less water should present less hydric stress to salamanders. We used 40 g of pure Agarose (VWR Life Science AMRESCO Agarose) per 1 L of water to make the salamanders models. We added black food coloring to mimic the dark color of most plethodontid species encountered during our surveys. The solution was heated to boiling, allowed to cool and then poured into a salamander mold made of latex rubber. The molds were constructed using a plastic replica of a Red‐cheeked Salamander (*Plethodon jordani*) available for purchase at the Great Smoky Mountains visitor centers. The plastic models are for the most part anatomically correct with slightly thicker limbs, which was ideal for demolding the agar models. To determine the temperature conditions of each microhabitat type, we placed a Thermochron iButton (Embedded Data Systems, DS1922L) underneath each agar model. The Thermochron iButton recorded temperature every 10 min, providing the approximate body temperature of a salamander occupying that microhabitat. The types of microhabitats used at each site depended on local availability but always included leaf litter, woody cover object, tree/shrub, and above ground. We deployed between 38–45 agar models (with Thermochrons) at each site, resulting in over 17,000 hr of measurements. The agar models weighed approximately 15.2 ± 1.7 g (mean ± *SD*) at the time of deployment. To estimate water loss for salamanders using each microhabitat type, we weighed agar models prior to deployment and reweighed them in the morning, afternoon, and night for three consecutive days. If at any point during their deployment a model lost more than 20% of its initial mass, we replaced it with a new model to ensure that the water loss rate was consistent. Models were weighed to the nearest 0.0001 g (Veritas—S123—Precision Balance). We calculated water loss as the mean change in mass of the agar models between successive measurement periods (mg/min). The water loss rate of our agar models was then used as a proxy for the moisture levels of the microhabitat. We also recorded the change in temperature of agar models (i.e., iButton data) between each weighing period.

### 2.4 Statistical analyses

All analyses were conducted using the R computing environment (Version 3.2.5) (R Core Development Team, 2016). To test whether temperatures and moisture conditions vary between microhabitat categories as well as differ spatially and temporally we categorized our sites as high (>1,100 m; mean = 1,332) or low elevation (<1,000 m; mean = 757). In addition, we noted the time each agar model was weighed: morning (4:00 a.m.–11:59 a.m.), afternoon (12:00 p.m.–7:59 p.m.), and night (8:00 p.m.–3:59 a.m.). We analyzed differences among microhabitats using univariate general linear mixed models as implemented in the “lme4” package. Each initial model included microhabitat type, elevation, and time of day as fixed effects including all possible interactions. We also included site and the date of sampling as random effects. We used separate analyses to examine how mean temperature, water loss rate, and temperature amplitude of microhabitats were affected by the predictor variables. We evaluated the contribution of each factor to the overall model using the ANOVA function in the “lmerTest” package and the Satterthwaite approximation of degrees of freedom to account for differences in sample variance. To make comparisons between the different levels of significant effects, we plotted the least square means and their standard error.

We used the survey data to assess salamander microhabitat use including potential interactions based on elevation and time of day. Some microhabitat categories had few observations, so we pooled these and retained only surface (e.g., any salamander on the surface of any substrate), under leaf litter, and under cover object for subsequent analyses. We used a generalized linear mixed model approach with a binomial distribution using the “lme4” package fitted with the presence/absence of a salamander as a response variable and microhabitat category, elevation, and time of day as fixed effects. We also include the microhabitat × time of day and microhabitat × elevation interactions. We included site and date of the census as random effects. We quantified whether salamanders used microhabitats at different frequencies by repeating the analysis using count data from the surveys, but using a Poisson distribution. As it is not possible to get a reliable estimate of the residual degrees of freedom for this analysis (Bolker et al., 2009), we made comparisons between microhabitats at different elevations and times of day solely by visually examining least square means and their standard errors generated from our model.

We also determined whether salamanders used microhabitats that differed in temperature and moisture levels compared to available microhabitats. We used a conditional logistic regression analysis using the clogit function in the “survival” package for this analysis. The conditional logistic regression examines matched pairs, which allows us to compare used habitat (presence) to available but unused habitat (absence) (Breslow & Day, 1980; Compton, Rhymer, & McCollough, 2002; Hosmer & Lemeshow, 1989). For each survey, we calculated the mean microhabitat temperature within 20 min from the start of the survey and mean water loss of the agar models during the relevant period during the day. We used the specific types of microhabitats used by salamanders to estimate mean salamander temperature and water loss values.

Finally, we used linear mixed models to determine whether: (a) microhabitats used by salamanders (presence plots) differed in temperature or potential water loss rates at high and low elevation, (b) presence microhabitats differed from absence microhabitats at each elevation, and (c) absence microhabitats differed between high and low elevation. We used separate models to explore variation in temperature and water loss by those predictors. We included site, date, time of day, and survey ID as random effects in these models.

## 3 RESULTS

### 3.1 Salamander microhabitat use varies across elevation and time of day

**Figure 1:**
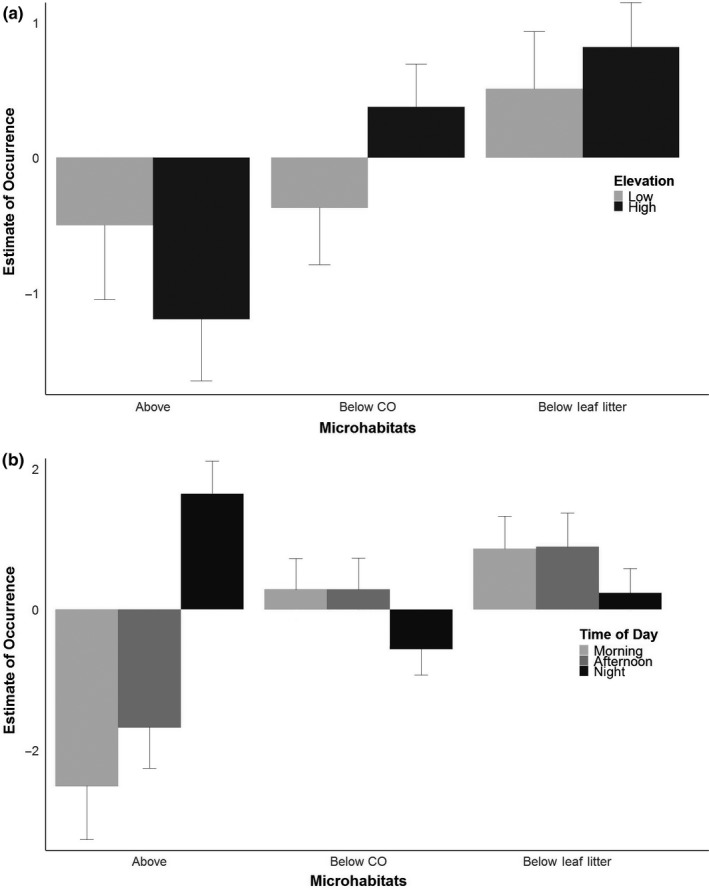
Least square means based on the generalized linear mixed effect model of salamander occurrence. There was a significant effect of the interaction between elevation and microhabitat type, and time of day and microhabitat type, but not for the full interaction term. (a) The least square means for the microhabitat and elevation interaction. (b) The least square means for the time of day and microhabitat interactions. Standard error bars are included. CO: cover object

In total, we completed 126 surveys and detected 389 plethodontid salamanders (Table 1). Our analysis of salamander occurrence and relative abundance data produced similar results. Based on occurrence data, salamanders were found under leaf litter most frequently at low‐elevation sites, whereas at high‐elevation sites salamanders were more likely to be found under cover objects or within the leaf litter (Figure 1a). However, more salamanders were captured under cover objects or leaf litter regardless of elevation (Figure 1a). At both elevations, salamanders were captured more frequently and in higher numbers above ground at night (Figure 1b). Whereas we detected no shifts in microhabitat use in the morning or afternoon, salamanders were less likely to be found under cover objects at night.

**Table 1:** Sample sizes (*N*) of salamander species captured during surveys. Mean elevation in meters ± standard errors (*SE*) is included when applicable

Species	*N*	Elevation (m) ± *SE*
*Desmognathus imitator*	3	810 ± 1
*Desmognathus monticola*	2	846 ± 0
*Desmognathus* sp.	15	1,150 ± 82
*Desmognathus wrighti*	45	1,115 ± 42
*Eurycea wilderae*	64	926 ± 24
*Plethodon jordani*	130	1,244 ± 26
*Plethodon jordani* x *teyahalee*	1	801
*Plethodon metcalfi*	35	1,256 ± 36
*Plethodon serratus*	37	1,058 ± 34
*Plethodon teyahalee*	56	1,071 ± 20
*Pseudotriton ruber*	1	789

Note

Several of the *Desmognathus* individuals found during the surveys were not identified to species as much of the study was conducted in area where identification is difficult and had no bearing on our study. We also found at least one hybrid individual, which exhibited traits of both *Plethodon jordani* and *P. teyahalee*.

### 3.2 Spatiotemporal variation in microhabitat temperature and moisture

**Figure 2:**
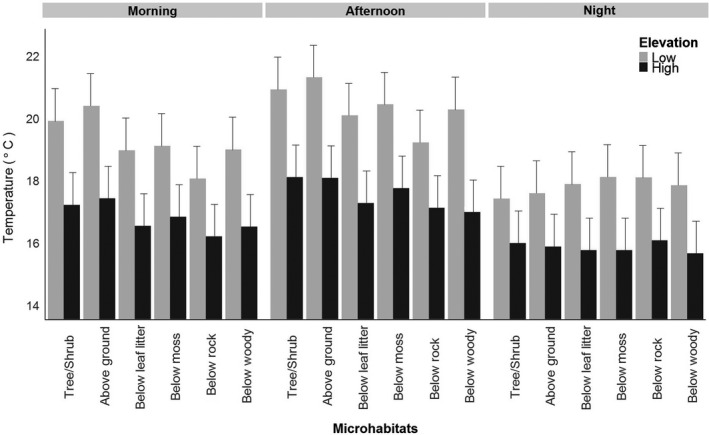
Least square means from a linear mixed effects model exploring variation in mean microhabitat temperature in our study. The analysis revealed a significant interaction between microhabitat type, time of day, and elevation. Standard error bars are included

**Figure 3:**
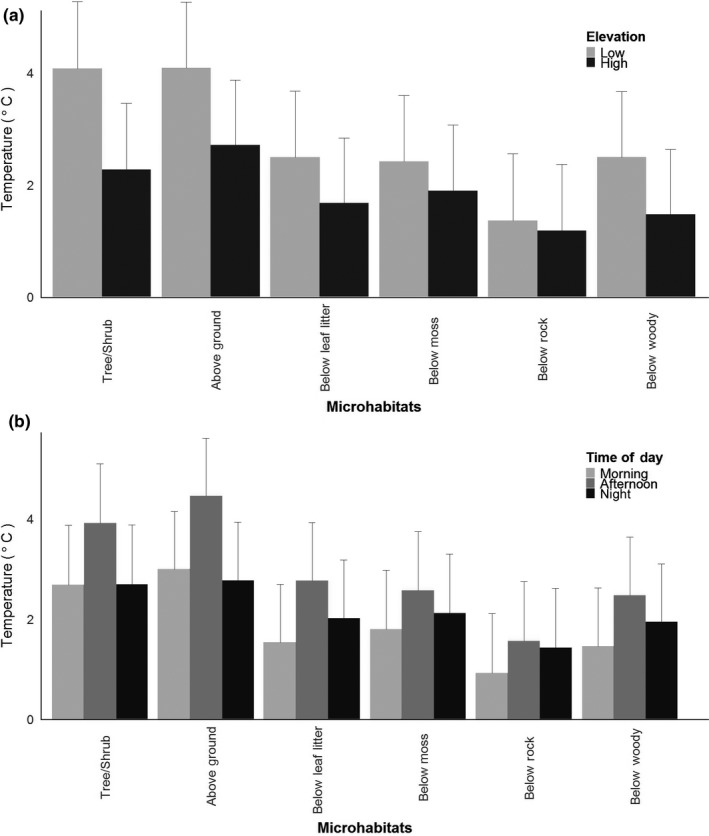
Least square means from a linear mixed effects model exploring variation in temperature amplitude in our study. There was a significant effect of the interaction between elevation and microhabitat type, and time of day and microhabitat type, but not for the saturated interaction term. (a) Least square means of temperature amplitude versus the interaction between microhabitat and elevation. (b) Least square means of temperature amplitude versus the interaction between time of day and microhabitat interactions. Standard error bars are included

Throughout the study, we obtained 106,893 records of microhabitat temperatures. A significant three‐way interaction term indicated that microhabitat temperature varies both spatially (across elevation) and temporally (across time of day) (Figure 2; Table 2). The microhabitat × elevation and microhabitat × time of day interactions were significant for both temperature range and water loss models (Table 2). Specifically, observed temperature range (i.e., the range between minimum and maximum temperatures) is greater at low elevation compared to high elevation for tree/shrub habitats, above ground, below leaf litter, and woody debris. However, the temperature range is similar at high and low elevation for the microhabitat categories of under moss and under rock (Figure 3a; Table 2). The temperature range is lowest during the morning period among all microhabitats, consistent with substrates not yet warming up in the sunlight. Temperature range was greatest during the afternoon in the tree/shrub and aboveground microhabitats compared to the morning and night hours; in other words, these substrates heated up the most during the day relative to other substrates (Figure 3b; Table 2). However, all belowground microhabitats had similar temperature ranges in the afternoon and at night, but temperature range was significantly lower in the morning.

**Table 2:** Results of linear mixed effects models comparing mean temperature, temperature range, and water loss rate among microhabitats, between elevations, time of day, and interactions when significant

Source	Sum of squares	*df*	*F*	*p*
Mean temperature (*σ* = 0.05)				
Microhabitat	0.77	5, 2,245.85	55.74	**<0.01**
Elevation	0.04	1, 5.66	12.60	**0.01**
Time of day	2.62	2, 2,245.1	473.13	**<0.01**
Microhabitat × elevation	0.03	5, 2,245.74	2.18	**0.05**
Microhabitat × time of day	0.56	10, 2,245.01	20.39	**<0.01**
Elevation × time of day	0.05	2, 2,245.32	8.74	**<0.01**
Microhabitat × elevation × time of day	0.08	10, 2,245.01	2.87	**0.01**
Temperature range (*σ* = 0.56)				
Microhabitat	182.37	5, 2,227.58	115.58	**<0.01**
Elevation	3.61	1, 1.89	11.43	0.08
Time of day	39.02	2, 2,226.28	61.83	**<0.01**
Microhabitat × elevation	4.93	5, 2,227.07	3.13	**<0.01**
Microhabitat × time of day	13.41	10, 2,226.03	4.25	**<0.01**
Agar model water loss rate (*σ* = 1.50)				
Microhabitat	865.96	6, 2,226.8	75.365	**<0.001**
Elevation	7.88	1, 2.7	3.43	0.171
Time of day	155.27	2, 2,226.3	33.782	**<0.001**
Microhabitat × elevation	120.5	5, 2,227.4	10.487	**<0.001**
Microhabitat × time of day	241.54	10, 2,226	10.511	**<0.001**

Note

We included site and date of sampling as random factors. Degrees of freedom (*df*) were calculated using a Satterthwaite approximation. The residual standard deviation (*σ*) is presented for each model. Significant *p* values are in boldface.

**Figure 4:**
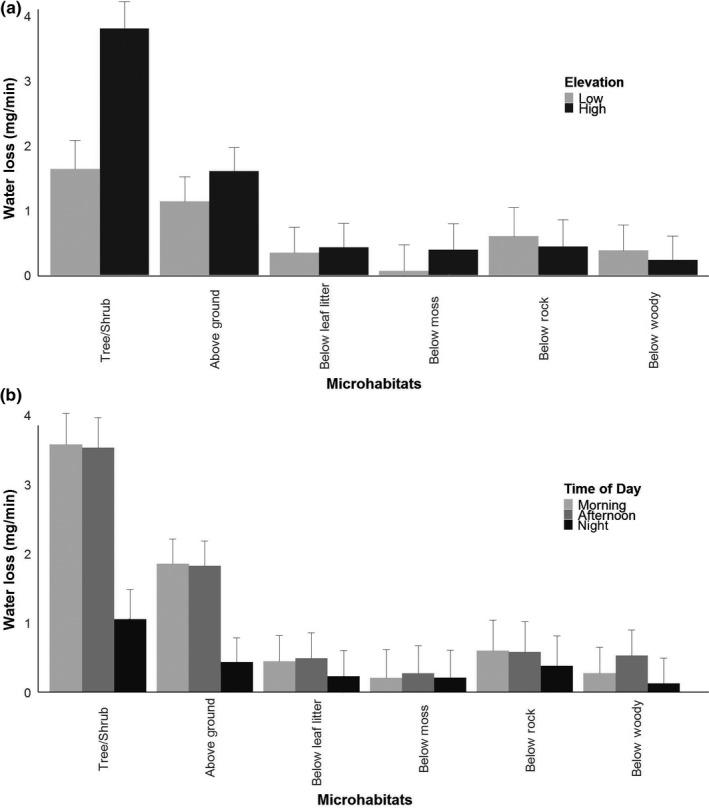
Least square means from a linear mixed effects model comparing rates of water loss against environmental factors. We observed significant interactions between elevation and microhabitat type and time of day and microhabitat type. We found no statistical support for the three‐way interaction term. (a) Least square means for the microhabitat and elevation interaction. (b) Least square means for the time of day and microhabitat interactions. Standard error bars are included

Overall, water loss rates differed by time of day but not by elevation (Figure 4a; Table 2). However, specific microhabitats did differ between elevations with arboreal microhabitats resulting in higher water loss rates for agar models compared to low‐elevation sites. Moreover, water loss rates were estimated to be similar throughout the day in subsurface microhabitats (Figure 4b; Table 2). However, rates of water loss for salamanders using aboveground microhabitats significantly exceeded subsurface values in the morning and afternoon. In other words, estimated salamander water loss rates varied spatially and temporally for certain microhabitat types; however, overall water loss rates only varied temporally and not spatially.

### 3.3 Salamander microhabitat use tracks both temperature and moisture

**Figure 5:**
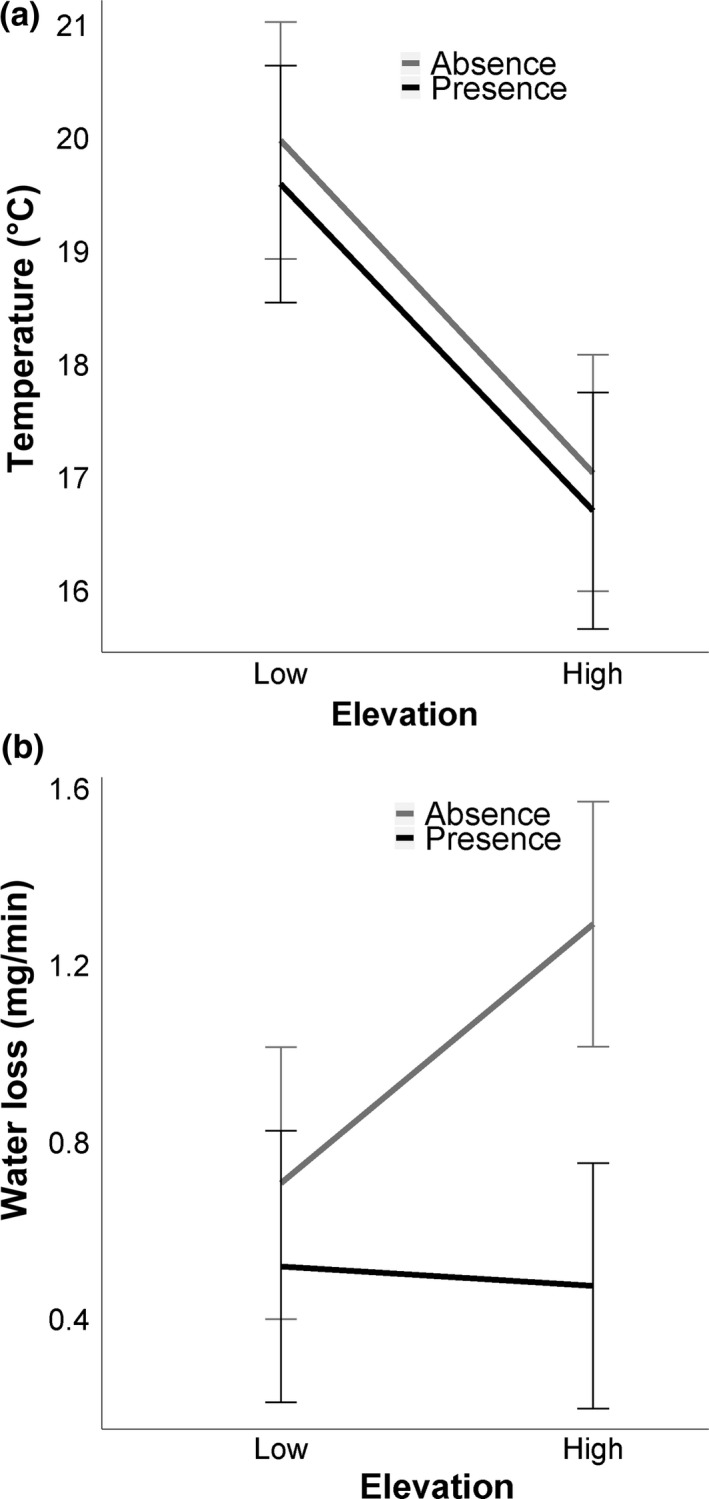
Least square means based on the linear mixed effect model of temperature (a) and water loss rates (b) separated by presence and absence microhabitats at both high and low elevation. Standard error bars are included

The conditional logistic regression revealed that salamanders preferred microhabitats with significantly lower temperature (odds ratio = 0.27, *p* < 0.001) and lower water loss rates (odds ratio = 0.39, *p* < 0.001). Both analyses suggest that salamanders are found in much higher frequency in cooler and wetter microhabitats. This contrasts with the unpaired analysis, which found no difference in temperature between presence and absence microhabitats but did reveal that temperature for both presence and absence microhabitats were lower at high‐elevation sites (Figure 5a; Table 3). In contrast, salamanders chose microhabitats that provided potential for significantly lower water loss rates compared to unused microhabitats at high‐elevation sites (Figure 5b; Table 3). Furthermore, water loss rates between high and low elevation were similar for microhabitats where salamanders were present (Figure 5b; Table 3).

**Table 3:** Results of linear mixed effects models comparing mean temperature and water loss rate

Source	Sum of Squares	*df*	*F*	*p*
Mean temperature				
Presence/absence	0.02	1, 141.18	6.32	**0.01**
Elevation	0.04	1, 4.317	13.68	**0.02**
Presence/absence × elevation	<0.01	1, 141.18	<0.01	0.99
Water loss				
Presence/absence	9.76	1,128.30	10.14	**<0.01**
Elevation	2.18	1, 150.39	2.27	0.13
Presence/absence × elevation	3.82	1, 128.30	3.97	**0.05**

Note

These models test for differences between occupancy of different microhabitats (e.g., Presence [used] and Absence [unused]), high and low elevation, and the interaction between these factors. Site, date, and time of day were included in the model as random effects. Degrees of freedom (*df*) were calculated using a Satterthwaite approximation. Significant *p* values are in boldface.

## 4 DISCUSSION

The Bogert effect is a pattern of physiological stasis due to regulatory behaviors. The behaviors enable a species to persist in changing environments but insulate physiological traits from the action of natural selection. However, the mechanisms that allow this to occur are still relatively unknown. This is particularly enigmatic for species that must face challenges regarding thermal and hydric balance, such as amphibians. Here, we have shown that salamanders simultaneously regulate both body temperature and moisture levels through the use of different microhabitats. Specifically, our analyses revealed differences in microhabitat use by salamanders that result in lower rates of water loss and access to cooler temperatures. Despite their consistent use of cooler microhabitats at all sites, temperature decreased with elevation, indicating that either thermal preferences decrease with elevation or that behavior is only partially effective at buffering environmental variation. In contrast, salamanders maintained similar rates of water loss across elevation. The stability in water loss rates could reflect better behavioral buffering by the salamanders but it could also reflect lower spatial variation in the hydric environment. Our data revealed limited differences in moisture conditions with elevation. However, we did detect diurnal fluctuations in moisture conditions. One potential caveat to our approach is that agar models do not always accurately reflect species‐specific rates of water loss rates in plethodontid salamanders (Riddell, Apanovitch, Odom, & Sears, 2017). Nevertheless, they do provide a reasonable estimate of moisture conditions that would impact the water loss rates of salamanders. Therefore, we used a combination of our data on habitat use, temperature, and approximate water loss in order to provide a similar assessment following Huey et al. (2003).

It is well known that salamanders use behavior to regulate temperature and moisture they experience over extended periods of time. Plethodontid salamanders will often spend months underground to escape warm and dry conditions, and they often retreat underground during the day when they are inactive before returning to the surface at night. However, once they are active, salamanders are limited in their ability to regulate temperature and moisture compared to non‐amphibian ectotherms, which may reduce the potential for behavioral compensation to buffer salamanders from climate change. Thermoregulation for other ectotherms typically involves multiple behavioral shifts throughout an activity period. For example, a heliothermic lizard can change perch sites between gradients of shade and sun in order to remain active. In contrast, a salamander is restricted in microhabitat options by the need for maintaining both strict temperature and moisture requirements. One strategy used by salamanders is to be nocturnal. However, our results demonstrate that salamanders can use behavior to regulate the temperature and moisture conditions they experience at a much smaller spatial and temporal scale. In light of climate change, this ability to further refine the environment they experience through microhabitat selection may be important for maintaining activity times. Specifically, by changing their microhabitat use throughout the day, they might be able to remain active whereas otherwise they might need to retreat during thermally unsuitable parts of the day.

Despite the substantial thermal variation that we observed across microhabitats, time of day, and elevation, we did not observe temperatures in excess of salamanders’ upper thermal limits. The values for presence and absence microhabitats both fall within the range of preferred temperatures for many species of plethodontid salamanders and none of the mean temperatures for any microhabitat at either elevation or time of day approached critical thermal maximums for any species (Spotila, 1972). This also explains why we do not see a more extreme difference in temperature between unused microhabitats and those used by salamanders. For example, Farallo and Miles (2016) found salamanders in West Virginia below leaf litter at 20°C when above‐litter temperatures exceeded 50°C. High temperatures, exacerbated by solar radiation, were likely mitigated by the thick layer of leaf litter between the animal and the surface. Of the 106,893 records of microhabitat temperatures, we noted only 14 instances of the temperature exceeding 30°C and 379 instances exceeding 25°C which account for 0.01% and 0.35% of all total measurements, respectively. Based on critical thermal maximum and thermal preference trials conducted by Spotila (1972) and Brattstrom (1963), both of which assessed several species of plethodontid salamanders from the eastern United States, thermal preferences for this group range between 12.0 to 25.6°C. Indeed, there is much less variation in critical thermal maximum which have values between 31.5–34.8°C. These data indicate that ambient temperatures rarely exceeded critical thermal thresholds for salamanders at any microhabitat or point in time during our study, and most microhabitats maintained a temperature preferred by many species of plethodontid. In fact, 89,204 (83.45%) of our recorded microhabitats temperatures were below 20°C, yet salamanders still consistently selected slightly cooler microhabitats. Furthermore, salamanders are active nearly all year in the Great Smoky Mountains National Park. This is unlike other regions in the eastern United States where salamander activity is frequently constrained by periods of warm and dry conditions. Thus, microhabitat selection may be less important for salamanders at our study sites than in other locations that experience more extreme conditions making our results conservative estimates of the ability of salamanders to select microhabitats.

Our data also highlights the importance of scale when addressing evolutionary and ecological questions. Whereas microhabitat temperatures varied both spatially (across elevation) and temporally (across time of day), microhabitat moisture varied only by time of day, with no additional effects of elevation. This indicates that the selective pressures influencing behavior differ between hydric and thermal environments. Importantly, a study conducted at a single elevation might conclude that the abiotic stressors shaping behavior are constant across traits. The expansion of a survey to include multiple localities would alter such a conclusion and would reveal evidence for heterogeneity in the responses of traits to environmental variation. More broadly, these results suggest that inferences of physiological and behavioral constraints differ across distinct geographic and phylogenetic scales. For example, in ground skinks from the Australian Wet Tropics, heat tolerance is predicted by microhabitat use, whereas cold tolerance and thermal optimum are predicted by elevation (Muñoz et al., 2016). In other words, the multiple selective factors that shape behavioral and physiological evolution often differ across distinct scales of analysis, highlighting the importance of integrating the multiple ecological effects that may simultaneously be at play.

We conclude that plethodontid salamanders use behavioral compensation in manner consistent with the Bogert effect. Nonetheless, the effectiveness of such behavioral buffering is likely to change as global climate change proceeds. Although the temperatures recorded in our study were well below the critical thermal maximum for most plethodontids, increases in temperature will result in increased rates of evaporation and water loss for all species in this diverse group. Hence, the use of behavioral buffering to avoid deleterious moisture conditions will likely become more challenging for salamanders as temperatures continue to rise and precipitation patterns are altered, the severity of this impact remains to be seen. Even in the Great Smoky Mountains National Park, soil water storage is expected to decrease, and evaporative deficit is predicted to increase despite potential increases in precipitation levels (Alder & Hostetler, 2013; McCabe & Wolock, 2011; Thrasher et al., 2013). In other areas of the eastern United States where habitats are less suitable or exhibit greater seasonality, we would expect climate change to pose a far greater problem for salamanders. As temperatures continue to rise around the world, the microhabitats used by salamanders will become less suitable and promote higher rates of desiccation, decreasing availability for salamander activity. If so, then the mechanisms by which salamanders regulate their temperature and moisture might also change.

Behavioral buffering involves changes to microhabitat use, which may result in evolutionary stasis of physiological processes. Specifically, if individuals across a species range consistently seek out specific temperatures and moisture levels then genetic variation to cope with altered environmental conditions may be lost over time. However, evolutionary shifts may occur on other traits correlated with activity such as morphology that allow for the use of different microhabitats that provide these constant environmental conditions. This has only been demonstrated so far in *Anolis* lizards (Muñoz & Losos, 2018), but the principle may be far reaching. We observed that salamanders change microhabitats to track lower temperatures and increased moisture. Under climate change scenarios this may include covariation with other traits resulting in indirect shifts in coloration and morphology as a consequence of a shift in microhabitat selection. Hence, behavior shifts may impart multiple, potentially conflicting evolutionary impacts, underscoring the importance of considering behavioral shifts in light of species’ multidimensional niches.

## CONFLICT OF INTERESTS

None declared.

## AUTHOR CONTRIBUTIONS

VRF and DBM conceived and designed the project. VRF and RW completed the field work. VRF completed the analyses. All authors contributed to the editing of the manuscript.

## DATA ACCESSIBILITY

Data available from the Dryad Digital Repository: https://doi.org/10.5061/dryad.73q5474.
